# An ampullary adenoma presenting with jaundice caused by duodenal intussusception: a case report

**DOI:** 10.1186/s40792-024-01822-z

**Published:** 2024-01-22

**Authors:** Hiroyuki Nojima, Hiroaki Shimizu, Mihono Hirota, Takashi Murakami, Masato Yamazaki, Kazuto Yamazaki, Kiyohiko Shuto, Chihiro Kosugi, Mikihito Mori, Akihiro Usui, Tetsutaro Sazuka, Keiji Koda

**Affiliations:** 1 Department of Surgery, Teikyo University Chiba Medical Center, Anesaki, Ichihara, Chiba 3426-3299-0011 Japan; 2 Department of Pathology, Teikyo University Chiba Medical Center, Anesaki, Ichihara, Chiba 3426-3299-0011 Japan

**Keywords:** Ampullary adenoma, Duodenal intussusception, Jaundice

## Abstract

**Background:**

Ampullary adenomas are premalignant lesions. However, biliary obstruction causing jaundice is rare. Duodenal intussusception secondary to an ampullary adenoma rarely occurs because of the fixed position of the duodenum in the retroperitoneum. Herein, we have described a rare case of ampullary adenoma with jaundice caused by duodenal intussusception.

**Case presentation:**

A 40-year-old woman presenting with vomiting and yellowish discoloration of the skin was admitted to another hospital. The patient had experienced recurrent epigastric pain and vomiting for the past 18 months. Blood test results showed elevated levels of bilirubin (3.9 mg/dL), and abdominal computed tomography (CT) showed a 60-mm hypovascular mass in the third part of the duodenum and a left lateral shift of the dilated common bile duct. The patient was referred to our hospital for further evaluation. She recovered from hyperbilirubinemia spontaneously (levels of bilirubin, 1.0 mg/dL), and the CT showed a tumor shift from the third part of the duodenum to the second part and improvement of the dilated common bile duct. Hypotonic duodenography revealed a tumor that moved easily from the second to the third portion of the patient's position. Upper gastrointestinal endoscopy revealed a large papillary tumor occupying the second part of the duodenum, which was diagnosed as an adenoma through biopsy. The possibility of malignancy could not be negated owing to the presence of jaundice and an elevated carbohydrate antigen 19-9 level (76.0 U/mL). Pancreaticoduodenectomy was performed. The resected specimen showed a 60 × 40 × 40-mm pedunculated ampullary mass with submucosal elongation. The pathological examination indicated that the ampullary tumor was a high-grade intestinal adenoma. The postoperative course was uneventful, and the patient was discharged 26 days postoperatively.

**Conclusions:**

This report describes a rare case of a patient with an ampullary adenoma presenting with jaundice resulting from duodenal intussusception. Owing to the possibility of a postoperative cancer diagnosis which may have caused the biliary obstruction and the difficulty in making an accurate preoperative diagnosis, it is imperative to choose the appropriate surgical procedure such as a pancreaticoduodenectomy.

## Background

Duodenal papillary adenomas have recently been classified as premalignant lesions in terms of carcinogenesis through an adenoma-carcinoma sequence [1]. However biliary obstruction leading to jaundice is a rare occurrence. Among all markers of ampullary tumors, jaundice is the strongest independent predictor of cancer [2]. Duodenal intussusception secondary to an ampullary adenoma rarely occurs because of the fixed position of the duodenum in the retroperitoneum. Current treatment options for ampullary tumors include endoscopic resection and surgical procedures including pancreaticoduodenectomy (PD), pancreas-preserving duodenectomy, and ampullectomy [3]. The accuracy of biopsy is relatively low, and in some cases, resected tumors diagnosed as papillary adenoma preoperatively are ultimately diagnosed as carcinoma of the adenoma or adenocarcinoma. Owing to the possibility of a postoperative diagnosis of cancer that may have led to preoperative bile duct obstruction and the presentation of jaundice, and the difficulty of accurate preoperative diagnosis, it is necessary to choose an appropriate surgical procedure, including PD. In this case report, we have described a rare case of a patient with an ampullary adenoma presenting with jaundice due to duodenal intussusception.

## Case presentation

**Fig. 1:**
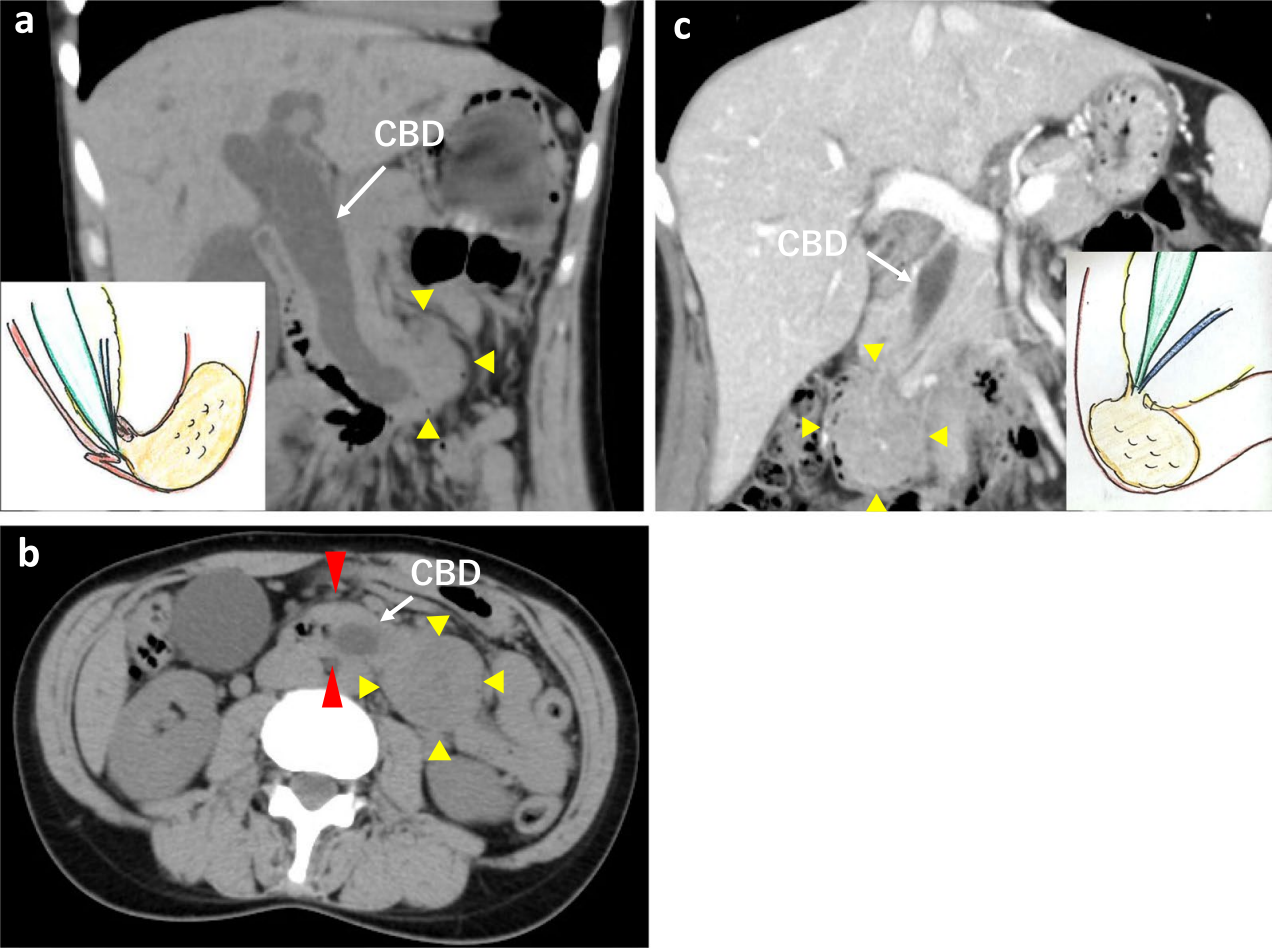
Contrast-enhanced CT. **a**, **b** A 60-mm hypovascular tumor (yellow arrowhead) in the third part of the duodenum and a left lateral shift of the dilated common bile duct (CBD). The obstruction was caused by duodenal wall ampulla-side partial intussusception (red arrowhead). **c** A 60-mm hypovascular tumor (yellow arrowhead) that shifted from the third part of the duodenum to the second part as well as improvement in the dilated common bile duct (CBD)

**Fig. 2:**
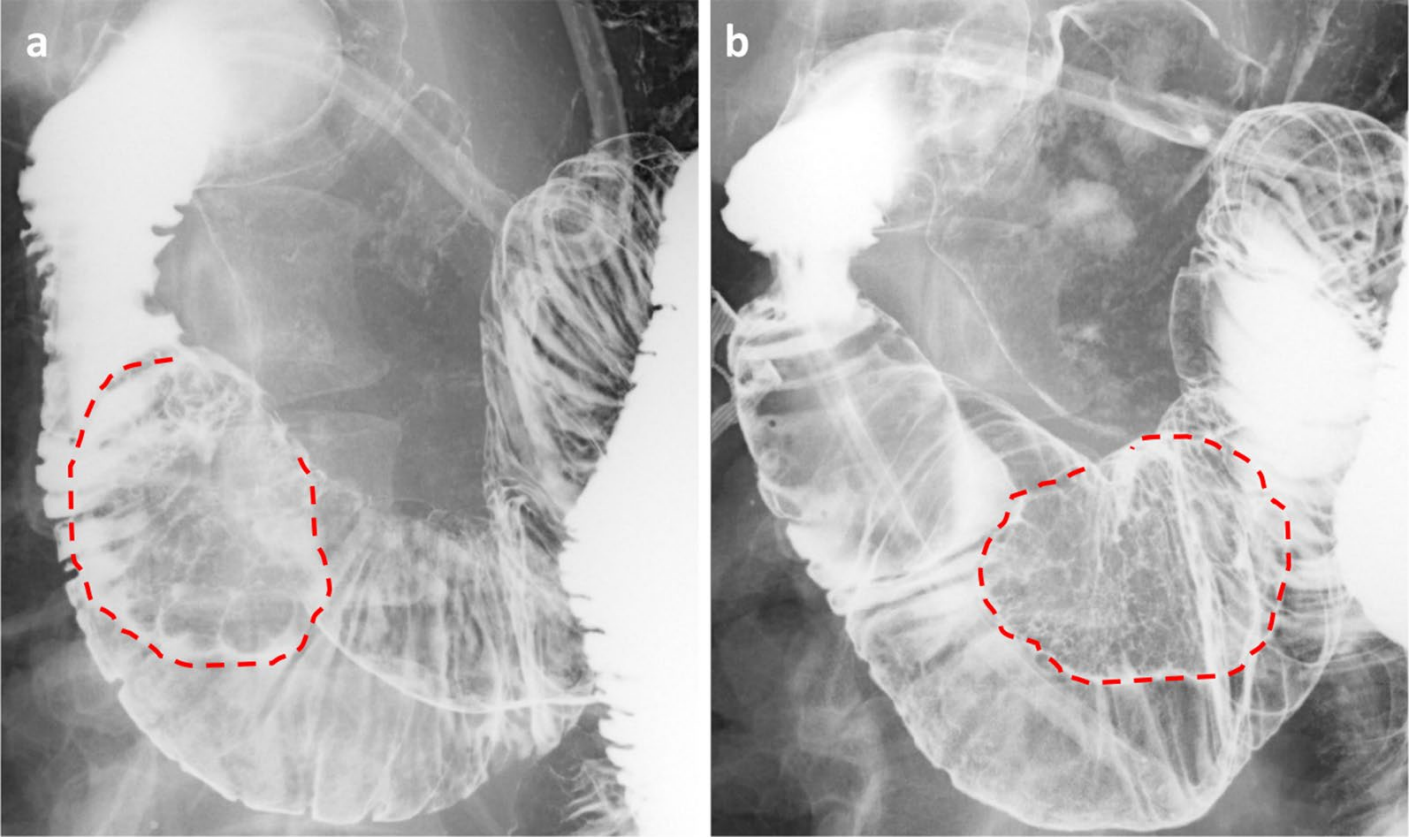
**a**, **b** Hypotonic duodenography. The tumor (red lines) was easily moved from the second to the third portion in the patient’s position.

**Fig. 3:**
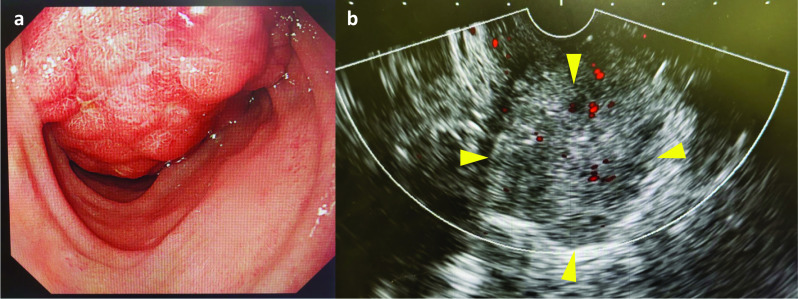
**a** Upper gastrointestinal endoscopy. A large papillary tumor (yellow arrowhead) occupying the second part of the duodenum. **b** Endoscopic ultrasonography. A hypoechoic ampullary mass (yellow arrowhead) showing no invasion of the surrounding organs such as the pancreas or bile ducts

**Fig. 4:**
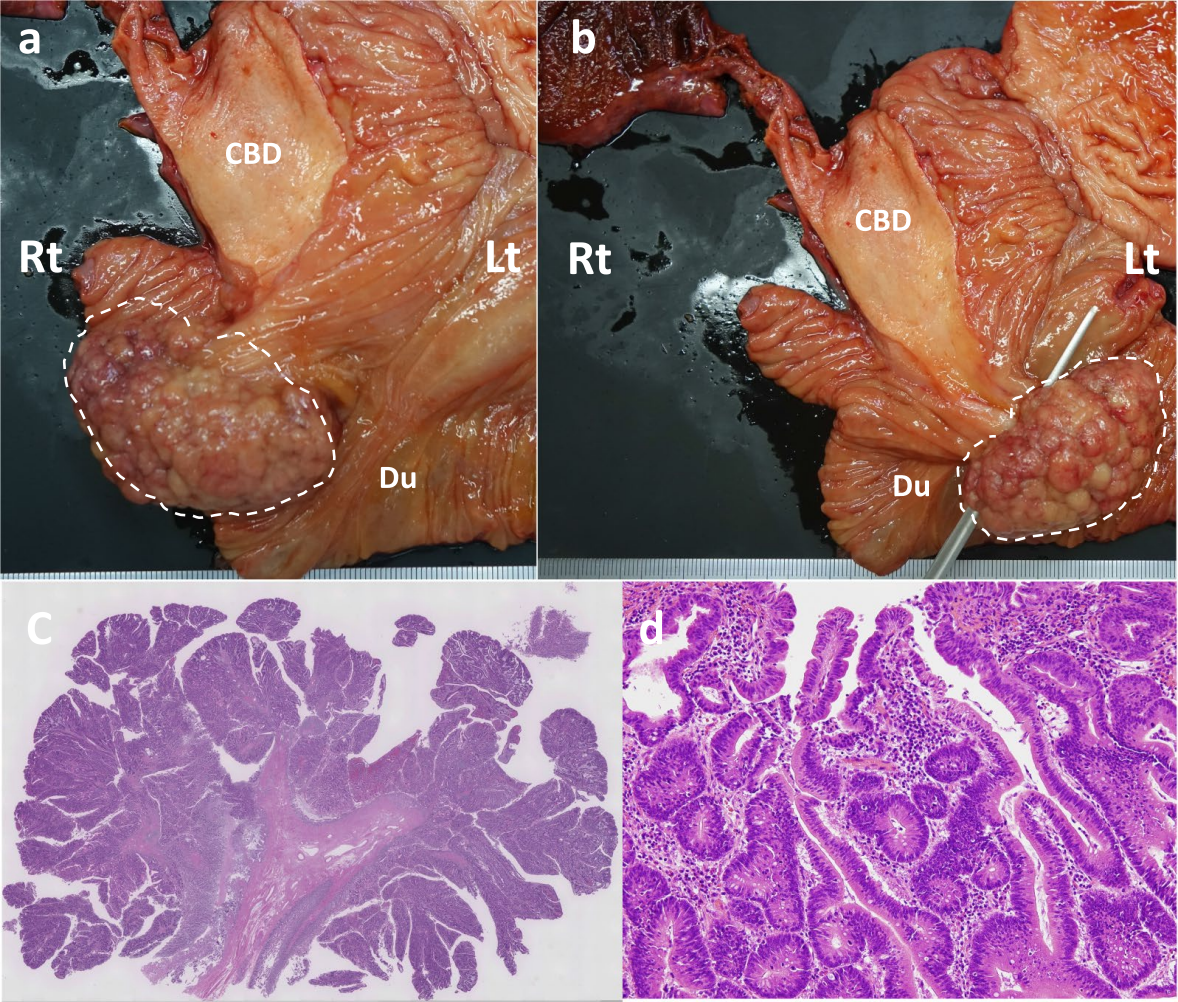
**a**, **b** (Rt: right; Lt: left) Resected specimens. A 60 × 40 × 40-mm pedunculated ampullary mass with submucosal elongation. *CBD* Common bile duct, *Du* duodenum. **c**,** d** (**c** ×12.5; **d** ×100) HE staining of the ampullary tumor. An intestinal-type adenoma of a high grade

A 40-year-old woman with vomiting and jaundice was previously admitted to another hospital. The patient had experienced recurrent episodes of epigastric pain and vomiting for the past 18 months. Her blood test results showed elevated levels of bilirubin (3.9 mg/dL), while abdominal computed tomography (CT) showed a 60-mm hypovascular mass in the third part of the duodenum and a left lateral shift of the dilated common bile duct (CBD), suggesting duodenal intussusception (Fig. 1a, b). The patient was referred to our hospital for further evaluation. She had a history of two cesarean sections but no relevant familial medical history. The patient recovered from the hyperbilirubinemia spontaneously and no endoscopic biliary drainage was performed. After admission to our hospital, the patient’s blood tests revealed a serum total bilirubin of 1.0 mg/dL, aspartate aminotransferase of 18 IU/L, alanine transaminase of 24 IU/L, alkaline phosphatase of 81 IU/L, and gamma-glutamyl transpeptidase of 99 IU/L. As for tumor markers, serum carcinoembryonic antigen and carbohydrate antigen (CA) 19-9 levels were 1.3 ng/mL and 76.0 U/mL, respectively. CT showed a 60-mm hypovascular tumor that had shifted from the third to the second part of the duodenum. Improvement in the dilated CBD was also observed (Fig. 1c). Hypotonic duodenography revealed a tumor that moved easily from the second to the third portion of the duodenum (Fig. 2). Upper gastrointestinal endoscopy revealed a large papillary tumor occupying the second part of the duodenum, which was diagnosed as a high-grade intestinal adenoma via biopsy, similar to the previous pathological results (Fig. 3a). Endoscopic ultrasonography revealed a hypoechoic ampullary mass that did not invade the surrounding organs such as the pancreas or bile ducts. (Fig. 3b). The possibility of malignancy could not be ruled out because of the large tumor size, even with multiple biopsies, as well as the elevated CA19-9 level (76.0 U/mL). After discussing the treatment strategy with the patient and her family, PD was performed. Laparotomy revealed that the ampullary tumor moved easily from the second to the third portion and the anatomical abnormality such as increased mobility of the second part of the duodenum along with the pancreatic head from the retroperitoneum was confirmed during the intraoperative Kocher maneuver. The operative time was 445 min, and 92 mL of blood was lost. The resected specimen showed a 60 × 40 × 40-mm pedunculated ampullary mass with submucosal elongation (Fig. 4a, b). The pathological examination indicated that the ampullary tumor was a high-grade intestinal adenoma (Fig. 4c, d). Immunostaining was negative for MUC2 and MUC5AC, while the median MIB-1 index was 30%. The postoperative course was uneventful, and the patient was discharged 26 days postoperatively. The patient was well with no signs of recurrence after 15 months postoperatively.

## Discussion

Recently, ampullary adenomas have been regarded as premalignant lesions in terms of carcinogenesis via the adenoma-carcinoma sequence. Ampullary adenomas, representing 5% of gastrointestinal neoplasms, are often asymptomatic and detected through upper endoscopy [4]. Symptoms of ampullary adenomas presenting with jaundice related to biliary obstruction caused by a tumor are rare [5, 6]. Hornick et al. reported that clinical jaundice was the strongest independent predictor of cancer among all ampullary tumors [2]. The authors also suggested that benign ampullary adenomas rarely present with biliary obstruction [2].

Duodenal intussusception secondary to an ampullary adenoma rarely occurs because of the fixed position of the duodenum in the retroperitoneum. In cases of intestinal malrotation, duodenal intussusception can occur because of the excessive mobility of the duodenal wall [7]. There are also a few reports on other factors that cause duodenal intussusception, such as duodenal tumor prolapse, duodenal diverticulum, and duplication cysts [8–10]. In this case, hypotonic duodenography revealed an ampullary mass that easily moved from the second to the third portion of the duodenum because of the increased mobility of the second part of the duodenum in this patient’s case. Normally, most of the duodenum, except for the bulbs, is fixed to the retroperitoneum, so duodenal intussusception is unlikely to occur. However, in this case, ampulla-side partial intussusception and biliary system deviation occurred, causing subsequent jaundice. This was probably due to the increased mobility of the second part of the duodenum along with the pancreatic head from the retroperitoneum. This anatomical abnormality was also confirmed during the intraoperative Kocher maneuver. The abdominal pain experienced by the patient for the past 18 months may have been due to repeated duodenal intussusception. Similar to our case, Hirata et al. reported repeated duodenal intussusception owing to a 45-mm ampullary adenoma [11] (Table 1).

**Table 1:** Summary of the characteristics of duodenal intussusception cases

Cases	Symptoms	Diameter of lesion (mm)	Causes of intussusception	Treatments
1 [7]	Epigastric pain, weight loss, anemia	90	Mobility of duodenal wall	Pancreaticoduodenectomy
2 [8]	Jaundice	40	Duplication cysts	Resection of cystic lesion
3 [9]	Abdominal pain, vomiting	None	Duodenal diverticulum	Resection of diverticulum
4 [10]	Abdominal pain	35	Duodenal tumor prolapse	Resection of tumor
5 [11]	Edema, malaise	45	Ampullary mass	Pancreaticoduodenectomy
Our case	Vomiting, jaundice	60	Ampullary mass with the elongated mucosa	Pancreaticoduodenectomy

Currently, the treatment options for duodenal papillary tumors include endoscopic resection and surgical procedures including PD, pancreas-preserving duodenectomy, and ampullectomy [3]. Regarding the endoscopic treatment of ampullary adenomas, the false-negative rate of endoscopic cancer biopsy has been reported to be 20–30% [12, 13]. The accuracy of biopsy is low, especially in large tumors, because the deep layers of the tumor are difficult to biopsy and cannot be diagnosed accurately. Resected cases preoperatively diagnosed as papillary adenomas were ultimately diagnosed as carcinomas of the adenoma or adenocarcinoma [14]. Among the 123 reported resected cases of duodenal papillary cancer, 23 cases of invasive adenocarcinoma were preoperatively diagnosed as benign or high-grade dysplasia [15]. Amini et al. also reported that lymph node metastases were detected in 22% of the 163 patients who underwent PD for stage T1 papillary carcinoma and recommended that surgical papillectomy without lymph node dissection should not be performed [16]. Regarding blood tests, elevated serum CA 19-9 levels are significantly associated with ampullary carcinoma [17]. We selected PD as the treatment of choice because of the possibility of a postoperative diagnosis of cancer which may have been the cause of the preoperative bile duct obstruction leading to jaundice.

Distinguishing between ampullary adenomas and adenocarcinomas is challenging, particularly in cases presenting with jaundice. In jaundiced patients with suspected ampullary adenocarcinoma, PD may be considered to achieve R0 resection and prevent the risk of recurrence in the case of adenocarcinomas.

## Conclusions

This case report describes a rare case of an ampullary adenoma with jaundice resulting from duodenal intussusception. Given the possibility of a postoperative cancer diagnosis that may have caused the preoperative bile duct obstruction and the difficulty in accurate preoperative diagnosis, it is necessary to choose an appropriate surgical procedure such as PD.

## Data Availability

Data will be made available by the corresponding author upon request.

## Abbreviations

PD: Pancreaticoduodenectomy
CT: Computed tomography
CBD: Common bile duct
CA: Carbohydrate antigen
